# Antiretroviral treatment response of HIV-infected children after prevention of mother-to-child transmission in West Africa

**DOI:** 10.7448/IAS.17.1.18737

**Published:** 2014-06-02

**Authors:** Camille Ndondoki, Fatoumata Dicko, Patrick Ahuatchi Coffie, Tanoh Kassi Eboua, Didier Koumavi Ekouevi, Kouakou Kouadio, Addi Edmond Aka, Karen Malateste, François Dabis, Clarisse Amani-Bosse, Pety Toure, Valériane Leroy, for the Paediatric IeDEA West African Working Group

**Affiliations:** 1 Inserm U897, ISPED, Université Bordeaux, Bordeaux, France; 2 Centre Inserm U897 “Epidémiologie & Biostatistiques”, ISPED, Université Bordeaux, Bordeaux, France; 3 Hôpital Gabriel Touré, Bamako, Mali; 4 IeDEA Regional Center, PACCI, CHU de Treichville, Abidjan, Côte d’Ivoire; 5 CHU de Yopougon, Abidjan, Côte d’Ivoire; 6 Centre Intégré de Recherches Biocliniques d’Abidjan, Abidjan, Côte d’Ivoire; 7 Centre de Prise en charge, de Recherche et de Formation (CePReF), Abidjan, Côte d’Ivoire; 8 Centre MTCT-plus, Abidjan, Côte d’Ivoire

**Keywords:** PMTCT, HIV, children, antiretroviral efficiency, West Africa

## Abstract

**Introduction:**

We assessed the rate of treatment failure of HIV-infected children after 12 months on antiretroviral treatment (ART) in the Paediatric IeDEA West African Collaboration according to their perinatal exposure to antiretroviral drugs for preventing mother-to-child transmission (PMTCT).

**Methods:**

A retrospective cohort study in children younger than five years at ART initiation between 2004 and 2009 was nested within the pWADA cohort, in Bamako-Mali and Abidjan-Côte d’Ivoire. Data on PMTCT exposure were collected through a direct review of children’s medical records. The 12-month Kaplan-Meier survival without treatment failure (clinical or immunological) was estimated and their baseline factors studied using a Cox model analysis. Clinical failure was defined as the appearance or reappearance of WHO clinical stage 3 or 4 events or any death occurring within the first 12 months of ART. Immunological failure was defined according to the 2006 World Health Organization age-related immunological thresholds for severe immunodeficiency.

**Results:**

Among the 1035 eligible children, PMTCT exposure was only documented for 353 children (34.1%) and remained unknown for 682 (65.9%). Among children with a documented PMTCT exposure, 73 (20.7%) were PMTCT exposed, of whom 61.0% were initiated on a protease inhibitor-based regimen, and 280 (79.3%) were PMTCT unexposed. At 12 months on ART, the survival without treatment failure was 40.6% in the PMTCT-exposed group, 25.2% in the unexposed group and 18.5% in the children with unknown exposure status (*p*=0.002). In univariate analysis, treatment failure was significantly higher in children unexposed (HR 1.4; 95% CI: 1.0–1.9) and with unknown PMTCT exposure (HR 1.5; 95% CI: 1.2–2.1) rather than children PMTCT-exposed (*p*=0.01). In the adjusted analysis, treatment failure was not significantly associated with PMTCT exposure (*p*=0.15) but was associated with immunodeficiency (aHR 1.6; 95% CI: 1.4–1.9; *p*=0.001), AIDS clinical events (aHR 1.4; 95% CI: 1.0–1.9; *p*=0.02) at ART initiation and receiving care in Mali compared to Côte d’Ivoire (aHR 1.2; 95% CI: 1.0–1.4; *p=*0.04).

**Conclusions:**

Despite a low data quality, PMTCT-exposed West African children did not have a poorer 12-month response to ART than others. Immunodeficiency and AIDS events at ART initiation remain the main predictors associated with treatment failure in this operational context.

## Introduction

At the end of 2010, an estimated 3.4 million children were living with HIV in the world, of whom 3.1 million in Africa. Furthermore, 330,000 children were newly infected with HIV in 2011, 43% fewer than the peak of 560,000 annual new infections observed in 2003 [1]. The most likely explanation was the scaling-up and effectiveness of interventions to prevent mother-to-child transmission (PMTCT) of HIV [2]. Although their relative efficacy is well documented, PMTCT antiretroviral drug regimens have raised many questions regarding antiretroviral drug resistance which could compromise the success of subsequent antiretroviral treatment (ART) of children [3,4]. Indeed, nevirapine (NVP) was so far the cornerstone of non-nucleoside reverse transcriptase inhibitor (NNRTI)-based ART, used in developing countries, in both adults and children. Nevirapine has been incorporated into both PMTCT (mostly as single-dose [sd]) and ART programmes.

Numerous studies have documented emergence and long-term persistence of HIV-1 nevirapine resistance mutations in both women and infants exposed perinatally to single-dose nevirapine (sd-NVP) for PMTCT [5–9]. The frequency of nevirapine resistance in the fourth week of life in children after PMTCT-based sd-NVP varied between 33 and 87% [8,10]. In Côte d’Ivoire, this frequency was, respectively, 23 and 6% in Agence Nationale de Recherche sur le SIDA et les Hépatites virales (ANRS) 1201/1202 Ditrame studies, where sd-NVP was associated with short-course zidovudine and short-course zidovudine and lamivudine, respectively [11,12]. A meta-analysis of nevirapine resistance provided pooled estimates of its prevalence [10]: 52.6% for children exposed to sd-NVP alone perinatally versus 16.5% when sd-NVP was combined with other antiretroviral drugs for PMTCT.

However, these studies did not appreciate the subsequent response to ART of HIV-infected children, an outcome that has been partly described so far elsewhere in Africa [13–16].

The cohort 1 of the P1060 randomized trial [14] showed that among African children with prior exposure to sd-NVP for PMTCT, subsequent ART consisting of zidovudine and lamivudine plus ritonavir-boosted lopinavir (LPV/r) resulted in better outcomes than treatment with zidovudine and lamivudine plus NVP. Indeed, 39.6% of children in the NVP arm were in virological failure by study week 24, compared to 21.7% in the LPV/r arm (*p=*0.02).

Thus, the 2010 guidelines for NVP-exposed infants advised that ART should be initiated with regimens based on LPV/r [17]. But there are many programmatic and operational obstacles to the use of protease inhibitor-based regimens in young children, because of unpleasant taste [18], mandatory refrigeration, interaction with co-treatment of tuberculosis [19], limitation for second line options and high cost of LPV/r.

In the Neverest randomized trial [16], children with prior exposure to sd-NVP, who initiated LPV/r-based treatment and achieved viral suppression (<400 copies/ml) for three or more months were randomized to either remain on LPV/r or switch to NVP. The reuse of NVP after achieving viral suppression with a LPV/r-based regimen resulted in lower rates of viremia greater than 50 copies/ml (Kaplan-Meier probability, 0.4; 95% CI: 0.3–0.5) than maintaining LPV/r regimen (0.6; 95% CI: 0.5–0.7), *p=*0.02. In these reports, the primary end-point was based on viremia. However, virological monitoring is not usually available under many operational circumstances, especially in West Africa where ART response of HIV-infected children after PMTCT exposure has not been described so far.

We aimed to assess the clinical and immunological response of HIV-infected children after 12 months on ART, according to their history of perinatal PMTCT exposure, in the paediatric IeDEA West African (pWADA) collaboration in Mali and Côte d’Ivoire.

## Methods

### Study design and population

The IeDEA West African data collection began in 2006, based on existing data issued from operational cohorts offering ART in 10 clinical centres in 7 countries in West Africa (Benin, Burkina Faso, Cote d’Ivoire, Gambia, Ghana, Mali and Senegal) that contribute standardized data to the pWADA collaboration [20,21]. All HIV-infected children under age 16, starting ART in these programmes from January 2000 to December 2009, were included. In 2010, a total of 2883 children under 15 at enrolment were included, of whom 1416 were younger than 5. The Mali and Cote d’Ivoire cohorts represented 74% of the cohort of children younger than five years at that time.

In these two latter countries, we nested a retrospective cohort in all HIV-infected children who initiated ART before their fifth birthday, between January 2004 and June 2009 and followed up in the five clinics contributing to the pWADA collaboration: four in Abidjan, Côte d’Ivoire (CEPREF, CIRBA, CHU Yopougon, MTCT+ Abobo-Avocatier) and one in Bamako, Mali (Gabriel Toure hospital).

These sites offered at that time a comprehensive HIV care and treatment programme serving children and family members, including PMTCT services, HIV diagnosis (PCR DNA for infant <18 months and serology for others), opportunistic infections prophylaxis, nutritional and community care, psychological care, and ART for children according to the World Health Organization (WHO) 2003 or 2006 recommendations or national guidelines [22,23].

During the inclusion period, the first-line ART regimen recommended for children was zidovudine or stavudine, lamivudine, and nevirapine or efavirenz by the national programmes in the two countries. But in Côte d’Ivoire, children with prior nevirapine exposure tended more often to be initiated on a LPV/r-based ART, especially when they have been included in a previous PMTCT trial.

For the present study, the retrospective and prospective collection of data was done using the IeDEA ethical comity clearance obtained by the national ethics review committees in each contributing country.

### Study monitoring and data collection

The inclusion date was the date of initiation of ART. Follow-up ended at death, at the last clinical visit prior to transfer to a different care provider or loss to follow-up, or 30 June 2010. If children withdrew from care or could not be located by three tracking attempts after a missed visit, with no clinical contact up to six months, they were classified as lost to follow-up.

At ART initiation, and every three months thereafter, children underwent clinical monitoring and education about adherence to treatment. Additional unscheduled visits were made by children needing acute care.

Clinical monitoring took into account all clinical events occurring after ART initiation, especially the occurrence of opportunistic infections and AIDS-defining clinical events. The following clinical events were particularly sought: severe malnutrition (<−3 standard deviation for weight-for-age z-score), toxoplasmosis, pulmonary tuberculosis, meningitis, oral candidiasis, Kaposi’s sarcoma, ear nose and throat infections, unexplained persistent diarrhoea (14 days or more), severe bacterial infections, chronic herpes simplex, pneumocystis pneumonia, severe pneumonia, HIV encephalopathy and severe sepsis.

CD4 cell counts and percentage were measured every six months at the National Reference Laboratory in Bamako and Abidjan. CD4 cell counts were measured by a dual-platform flow cytometry technique with an automated blood cell counter (MaxM, Beckman Coulter, Miami, FL, USA) at the screening visit, then six and twelve months after ART initiation. Viral load was not routinely assessed because of the frequent lack of availability of the assay.

Clinical and laboratory data were documented by physicians during patient visits using standardized forms. They were updated in the IeDEA paediatric database every six months. For this study, information on the PMTCT exposure was updated through a direct review of children’s medical records. When exposure to PMTCT intervention was documented, we investigated whether NVP had been used or not. Children were assigned as PMTCT-exposed or unexposed if this information was specified in the medical record, and PMTCT exposure was considered unknown otherwise.

The following baseline information was collected: date of birth, sex, clinical centre, date of ART initiation, weight, cotrimoxazole prophylaxis, CD4 count and per cent, WHO staging 3 or 4 event, and first-line ART regimen. Additionally, we collected information about CD4 count and per cent, WHO staging 3 or 4 event, and ART regimen for the six- and twelve-month visits and the date of latest news.

### Study end-points

The primary study objective was to compare the 12-month rate of clinical and immunological failure in HIV-infected children treated by ART, according to their PMTCT exposure. Four outcomes were analyzed: overall survival, survival without clinical event, survival without immunological failure, and survival without treatment failure (clinical or immunological failure).

In the absence of adherence data, we made the assumption that children enrolled in this cohort were adherent to ART. Clinical failure was defined as the appearance or reappearance of WHO clinical stage 3 or 4 events or any death occurring within the first 12 months of ART. Immunological failure was defined according to the WHO age-related immunological thresholds for severe immunosuppression [24].

### Statistical analysis

Descriptive statistics were presented as median values with inter-quartile ranges (IQR) for continuous variables. Categorized variables were presented as number or proportions (%) with their 95% confidence intervals. Chi-square test or Fischer exact test were used to compare baseline categorical variables and Kruskal-Wallis test was used to compare medians of baseline continuous variables. The z-scores of weight for age (WAZ) were calculated using SAS programme based on the Centers for Disease Control (CDC) 2000 growth reference year.

The 12-month probabilities of survival were computed using the Kaplan-Meier estimates for all children. The Log-rank test was used to compare survival curves according to PMTCT exposure categories.

Baseline factors associated with treatment failure at 12 months after ART initiation were analyzed using univariate analysis and then a multivariate Cox proportional-hazards model. Variables with *p*≤0.20 in the univariate analysis were included in a full multivariate analysis. A reduced adjusted model was ultimately created in which variables were excluded using a backward procedure.

The main analysis was conducted in the overall cohort and the secondary one compared the two known cohorts for PMTCT exposure.

To understand whether missing data for PMTCT exposure had introduced selection bias in our analysis, we performed a multivariate sensitivity analysis with the hypothesis of maximum bias. First, all children with unknown exposure to PMTCT were assigned to the PMTCT-exposed group. Then, they were assigned to PMTCT-not-exposed group.

All statistical analyses were assessed for statistical significance at the *p*<0.05 threshold and were performed using SAS 9.01 software.

## Results

### Study cohort

The retrospective data collection was carried out for the 1035 eligible children younger than five years at ART initiation (48% in Mali); PMTCT exposure was documented for only 353 children (34%) of whom 73 were exposed and 280 unexposed, and it remained unknown for 682 children (66%). Although the unknown cohort appears similar to the unexposed cohort, the characteristics of the three cohorts were different, except for sex (Table 1). Thus, the analysis of outcomes according to PMTCT exposure was performed among all children, in order to present a “real-life” cohort.

**Table 1:** Baseline characteristics of HIV-infected children at ART initiation according to PMTCT prophylaxis exposure, IeDEA paediatric West African Database on AIDS, Côte d’Ivoire and Mali, 2004 to 2009, (*N=*1035)

Children characteristics		Exposed to PMTCT, *N=*73	Unexposed to PMTCT, *N=*280	Unknown PMTCT exposure, *N=*682	*p*-value, exposed vs. unexposed	*p*-value, all compared
Country	*N* (%)				0.007	<0.0001
Cote d’Ivoire		60 (82.2)	185 (66.1)	287 (42.1)		
Mali		13 (17.8)	95 (33.9)	395 (57.9)		
Sex	*N* (%)				0.14	0.21
Girl		39 (53.4)	123 (43.9)	291 (42.7)		
Boy		34 (46.6)	157 (56.1)	391 (57.3)		
Median age (months) [IQR]	*N* [IQR]	11 [8–23]	27 [18–39]	29 [19–44]	<0.0001	<0.0001
Age class (months)	*N* (%)				<0.0001	<0.0001
<12		38 (52.0)	31 (11.1)	72 (10.6)		
12 to 36		28 (38.4)	162 (57.8)	359 (52.6)		
36 to 60		7 (9.6)	87 (31.1)	251 (36.8)		
Immunodeficiency*	*N* (%)				0.41	0.05
No		20 (27.4)	64 (22.9)	209 (30.7)		
Yes		53 (72.6)	216 (77.1)	473 (69.3)		
WAZ	*N* (%)				0.62	0.009
≥ − 3 SD		43 (58.9)	156 (55.7)	317 (46.5)		
<−3 SD		30 (41.1)	124 (44.3)	365 (53.5)		
WHO stage (3/4)	*N* (%)				0.0002	<0.0001
No		20 (27.4)	29 (10.4)	35 (5.1)		
Yes		53 (72.6)	251 (89.6)	647 (94.9)		
Cotrimoxazole prophylaxis	*N* (%)				0.41	0.02
No		10 (13.7)	29 (10.4)	45 (6.6)		
Yes		63 (86.3)	251 (89.6)	637 (93.4)		
First-line regimen	*N* (%)				<0.003	<0.0001
NNRTI-based		25 (34.2)	157 (56.1)	435 (63.8)		
PI-based		45 (61.7)	114 (40.7)	224 (32.8)		
3 NRTI		3 (3.1)	9 (3.2)	23 (3.4)		

PMTCT: prevention of mother-to-child transmission; WAZ: weight for age z-score; ART: antiretroviral treatment; PI: protease inhibitor; NNRTI: non-nucleoside reverse transcriptase inhibitor.

* Immunodeficiency defined according to the 2006 WHO recommendations: <25% or 1500 CD4 cells in those <12 months of age; <20% or 750 CD4 cells in those aged from 12 months to 35 months; <15% or 350 CD4 cells in those younger than five years.

### Baseline children characteristics

The median time to HIV diagnosis for the 1035 children was 23 months (IQR: [14 to 35 months]), and their median age at ART initiation was 27 months (IQR: [17 to 41 months]). Of these, 453 (43.7%) were females. Their median CD4% at ART initiation was 15.1% (IQR: [10.0 to18.0%]) and 91.9% of them presented WHO clinical stage 3 or 4. The first-line ART regimen was based on NNRTI drugs (nevirapine or efavirenz) for 617 (59.6%) children, on protease inhibitor for 383 (37.0%) children, and three NRTIs for 35 (3.4%) children. PMTCT-exposed children were mainly treated by a protease inhibitor-based regimen (61.7%). Children characteristics at ART initiation according to their PMTCT exposure are summarized in Table 1.

The baseline characteristics of PMTCT-exposed children differed from those unexposed and those who had unknown exposition status (Table 1): PMTCT-exposed children were significantly more often from Côte d’Ivoire, and were significantly younger at ART initiation (median age of 11 months vs. 27 months and 29 months, respectively; *p*<0.0001). They were less often at WHO clinical stage 3 or 4 (72.6% vs. 89.6% and 94.9% respectively; *p*<0.0001). Finally, they were less often treated by a NNRTI-based regimen (34.2% vs. 56.1% and 63.8%, respectively; *p*<0.0001).

### Clinical and immunological failure to ART according to PMTCT exposure

Among the 1035 children, 89 (8.6%) died during the first 12 months on ART, including 6 (8.2%) in the exposed group, 22 (7.8%) in the unexposed group, and 61 (8.9%) in the unknown exposure group (Table 2). Of these, 69 (77.5%) deaths of unknown reasons occurred during the first six months of treatment.

**Table 2:** Kaplan-Meier estimates of clinical and immunological failures according to PMTCT exposure after 12 months of ART

	Exposed to PMTCT (*N=*73)			Unexposed to PMTCT (*N=*280)			Unknown PMTCT exposure (*N=*682)				
	
Outcomes	*n* (%)	Free survival probability (%)	95% CI	*n*	Free survival probability (%)	95% CI	*n*	Free survival probability (%)	95% CI	*p*-value, exposed vs. unexposed (Log-Rank)	*p*-value, all compared (Log-Rank)
Deaths	6 (8.2)			22 (7.8)			61 (8.9)			0.91	0.80
6-month		93.0	[84.1–97.1]		93.6	[89.9–95.9]		92.1	[89.7–93.9]		
12-month		91.6	[82.2–96.1]		91.5	[87.4–94.3]		90.2	[87.6–92.3]		
Death and lost to follow-up	8 (11.0)			49 (17.5)			141 (20.6)			0.17	0.10
6-month		93.0	[84.1–97.1]		87.0	[82.5–90.5]		82.9	[79.9–85.6]		
12-month		88.8	[78.9–94.2]		82.2	[77.1–86.2]		78.9	[75.6–81.8]		
AIDS-related clinical events/ WHO stage (3 or 4)	29 (39.7)			140 (50.0)			381 (55.9)			0.01	0.0001
6-month		75.8	[63.5–84.4]		58.4	[51.9–64.3]		45.2	[40.9–49.3]		
12-month		63.4	[50.5–73.8]		44.7	[38.3–50.9]		32.5	[28.6–36.5]		
Clinical failure*	35 (47.9)			159 (56.8)			439 (64.4)			0.02	0.0001
6-month		70.5	[58.4–79.7]		54.9	[48.6–60.7]		41.5	[37.5–45.4]		
12-month		59.0	[46.6–69.4]		41.2	[35.1–47.2]		29.4	[25.8–33.1]		
Immunological failure*	23 (31.5)			102 (36.4)			201 (29.5)			0.19	0.12
6-month		72.9	[60.5–81.9]		67.4	[61.1–72.9]		73.7	[69.9–77.2]		
12-month		66.7	[53.9–76.6]		57.8	[51.2–63.8]		65.3	[61.1–69.1]		
Treatment failure*	47 (64.4)			198 (70.7)			507 (74.3)			0.02	0.002
6-month		52.2	[40.1–63.0]		38.2	[32.2–44.1]		31.1	[27.5–34.8]		
12-month		40.6	[29.1–51.8]		25.2	[20.0–30.7]		18.5	[15.5–21.7]		

* See definitions in the text; ART: antiretroviral treatment; PMTCT: prevention of mother-to-child transmission; CI: confidence limits intervals. IeDEA paediatric West African Database on AIDS, Côte d’Ivoire and Mali, 2004 to 2009, (*N=*1035).

The 12-month probability of survival after ART initiation did not differ between the exposed and other children (91.6% for exposed vs. 91.5% for unexposed and 90.2% for unknown exposure; Log-rank test: *p=*0.80) (Table 2).

The probability of being followed up at 12 months after ART initiation was 88.8% in the exposed group vs. 82.2% in the unexposed group and 78.2% in the unknown exposure group (Log-rank test: *p=*0.10).

In total 550 (53.1%) children had at least one AIDS defining event (WHO clinical stage 3 or 4) during the first 12 months of ART, with the most common event being malnutrition. The 12-month probability of not progressing to an AIDS clinical event (WHO stage 3 or 4) was 63.4% in the exposed group vs. 44.7% in the unexposed group and 32.5% in the unknown exposure group (Log-rank test: *p=*0.0001).

The 12-month probability of survival without clinical failure was significantly higher in the exposed children compared to the unexposed and unknown exposure ones (59.0% vs. 41.2% and 29.4%, respectively; Log-rank test: *p=*0.0001).

Out of the 326 (31.5%) children who experienced an immunological failure during their first 12 months on ART, 31.5% were in the exposed group, 36.4% in the unexposed group and 29.5% in the unknown exposure group. The probability of survival without immunological failure at 12 months after ART initiation was not different between the exposed children and other children (66.7% vs. 57.8% and 65.3%, respectively; Log-rank test: *p=*0.12).

Overall, a total of 752 (72.6%) children experienced a treatment failure (clinical or immunological) within the first 12 months on ART with a significant difference between the three groups. The probability of survival without treatment failure at 12 months after ART initiation was 40.6% in the exposed children vs. 25.2% in the unexposed children and 18.5% in the unknown exposure group (Log-rank test, *p=*0.002).

### Factors associated with ART failure within the first 12 months of ART in the whole cohort

In the univariate analysis, unexposed children (hazard ratio [HR] 1.4; 95% CI [1.0–1.9]) and children with unknown PMTCT exposure (HR 1.5; 95% CI [1.2–2.1]) were significantly more likely to be in the treatment failure than PMTCT-exposed children (*p=*0.01) (Table 3). But this assertion was no longer statistically significant in the adjusted analysis (adjusted hazard ratio [aHR]: 1.2; 95% CI [0.9–1.7] and aHR: 1.3; 95% CI [0.9–1.8] respectively; *p=*0.15). In the final adjusted analysis, the three factors associated with treatment failure after 12 months on ART were immunodeficiency at baseline (aHR 1.6; 95% CI: 1.4–1.9; *p=*0.001), having an AIDS clinical events at baseline (aHR 1.4; 95% CI: 1.1–1.9; *p=*0.02), and receiving HIV care in Mali compared to Côte d’Ivoire (aHR 1.2; 95% CI [1.0–1.4]; *p=*0.04).

**Table 3:** Factors associated with ART failure of HIV-infected children after 12 months of ART (cox model), IeDEA paediatric West African Database on AIDS, Côte d’Ivoire and Mali, 2004 to 2009, (*N=*1035)

Baseline characteristics	*N=*1035	Univariate model, HR [CI _95%_]	*p*	Adjusted model, aHR [CI _95%_]	*p*
Country			0.01		0.04
Cote d’Ivoire	532	1		1	
Mali	503	1.2 [1.0–1.4]		1.2 [1.0–1.4]	
Sex			0.6		
Girl	453	1			
Boy	582	0.9 [0.8–1.1]			
Age			0.7		
<12 months	141	1			
12 to 36 months	549	1.0 [0.8–1.3]			
36 to 59 months	345	0.9 [0.7–1.2]			
PMTCT exposure			0.01		0.15
Exposed	73	1		1	
Unexposed	280	1.4 [1.0–1.9]		1.2 [0.9–1.7]	
Unknown	682	1.5 [1.2–2.1]		1.3 [0.9–1.8]	
Immunodeficiency for age*					
No	293	1	0.0001	1	0.001
Yes	742	1.6 [1.3–1.8]		1.6 [1.4–1.9]	
AIDS-related clinical events/WHO stage (3 or 4)			0.0001		0.02
No	84	1		1	
Yes	951	1.8 [1.3–2.4]		1.4 [1.1–1.9]	
Cotrimoxazole prophylaxis			0.02		0.16
No	84	1		1	
Yes	951	1.3 [1.0–1.8]		1.2 [0.9–1.6]	
NNRTI-based regimen			0.8		
No	418	1			
Yes	617	1.0 [0.8–1.2]			

ART: antiretroviral treatment; HR: hazard ratio; aHR: adjusted hazard ratio; PMTCT: prevention of mother-to-child transmission; NNRTI: non-nucleoside reverse transcriptase inhibitor.

* Immunodeficiency defined according to the 2006 WHO recommendations: <25% or 1500 CD4 cells in those <12 months of age; <20% or 750 CD4 cells in those aged from 12 months to 35 months; <15% or 350 CD4 cells in those <5 years.

Among the children who experienced a treatment failure in the first 12 months of ART: 392 (52.1%) lived in Mali and 360 (47.9%) in Cote d’Ivoire. Among the 383 children who received LPV/r as first-line treatment, 320 (83.6%) lived in Cote d’Ivoire and 63 (16.5%) in Mali, in relation with a higher access to LPV/r offered in Côte d’Ivoire. However, we did not observe a statistical difference in the occurrence of treatment failure in children treated by LPV/r between these two countries (76.9% in Côte d’Ivoire vs. 76.2% in Mali, *p=*0.90). In fact, the children who received LPV/r in these sites were precisely those who were more severely ill at ART initiation, thus, with a high risk of treatment failure, and LPV/r treatment is more a marker of the advanced HIV disease.

The sensitivity analysis conducted showed that PMTCT exposure was not associated with treatment failure, when all unknown exposure children were assigned as exposed (aOR: 1.0 [0.9–1.2]; *p=*0.58), and when all unknown exposure children were assigned as unexposed (aOR: 1.3 [0.9–1.8]; *p=*0.07).

### Factors associated with ART failure within the first 12 months of ART in the cohorts with known PMTCT exposure

In adjusted analysis, a PMTCT exposure was not significantly associated with treatment failure (aHR 0.8; 95% CI [0.6–1.2]; *p=*0.3) when comparing the two cohorts with a known PMTCT exposure (Table 4). The 12-month treatment failure was correlated with immunosuppression (aHR 1.7; 95% CI [1.2–2.4]; *p=*0.001), and AIDS clinical events (aHR 1.7; 95% CI [1.1–2.7]; *p=*0.001) at ART initiation (Table 4).

**Table 4:** Factors associated with ART failure of HIV-infected children after 12 months of ART (cox model), IeDEA paediatric West African Database on AIDS, Côte d’Ivoire and Mali, 2004 to 2009, (*N=*353)

Baseline characteristics	*N=*353	Univariate HR [CI _95%_]	*p*	Adjusted aHR [CI _95%_]	*p*
Countries			0.03		0.14
Cote d’Ivoire	245	1		1	
Mali	108	1.3 [1.0–1.7]		1.2 [0.9–1.6]	
Sex			0.72		
Girl	162	1			
Boy	191	0.9 [0.7–1.2]			
Age			0.95		
<12 months	69	1			
12 to 36 months	190	1.0 [0.7–1.4]			
36 to 59 months	94	0.9 [0.6–1.4]			
PMTCT exposure			0.04		0.34
Not exposed	280	1		1	
Exposed	73	0.7 [0.5–0.9]		0.8 [0.6–1.2]	
Immunodeficiency for age *					
No	84	1	0.0005	1	0.001
Yes	269	1.7 [1.3–2.5]		1.7 [1.2–2.4]	
AIDS-related clinical events/WHO stage (3 or 4)			0.0003		0.001
No	49	1		1	
Yes	304	2.2 [1.4–3.3]		1.7 [1.1–2.7]	
Cotrimoxazole prophylaxis			0.04		0.10
No	39	1		1	
Yes	314	1.6 [1.0–2.4]		1.4 [0.9–2.2]	
NNRTI-based regimen			0.8		
No	171	1			
Yes	182	1.0 [0.8–1.3]			

ART: antiretroviral treatment; HR: hazard ratio; aHR: adjusted hazard ratio; PMTCT: prevention of mother-to-child transmission; NNRTI: non-nucleoside reverse transcriptase inhibitor.

* Immunodeficiency defined according to the 2006 WHO recommendations: <25% or 1500 CD4 cells in those <12 months of age; <20% or 750 CD4 cells in those aged from 12 months to 35 months; <15% or 350 CD4 cells in those <5 years.

## Discussion

This cohort study represents, to our knowledge, the first description of the ART response in HIV-infected children according to their PMTCT prophylaxis exposure under field circumstances in West Africa, a region with the lowest coverage of PMTCT programmes in Africa. Our study showed first that only 34% of the whole cohort had a documented PMTCT exposure, of whom only 21% were exposed. Second, in the context where only clinical and immunological monitoring is available, the risk of treatment failure was high overall, varying from 60 to 82% after 12 months on ART. Third, PMTCT exposure was not associated with treatment failure in the adjusted multivariate analysis nor in the whole cohort analysis, or the analysis restricted to the two cohorts with documented PMTCT exposure. In both analyses the strongest associated factors of treatment failure in children were AIDS clinical events and immunodeficiency at ART initiation. The empirical evidence we provide can inform similar treatment programmes in many parts of Africa.

So far, four studies have evaluated the ART response based on virological criteria according to the PMTCT exposure. The Ugandan observational cohort [13], compared the response to a NVP-based regimen in HIV-infected children exposed and unexposed to sd-NVP at birth, and showed that after 48 weeks of ART, 76% of the NVP-exposed and 80% of those not exposed children had a median viral load of <400 copies/ml (*p=*0.74). In Botswana, the Mashi randomized clinical trial [15] showed that virological failure by the six-month visit after ART initiation occurred in significantly more infants who had received a sd-NVP at birth than in infants who had received placebo (76.9% vs. 9.1%; *p*<0.001). Maternal and infant findings did not change qualitatively by 12 and 24 months after the initiation of ART. The cohort 1 of the P1060 randomized trial [14] showed that among children with prior exposure to sd-NVP for PMTCT, subsequent ART consisting of zidovudine and lamivudine plus LPV/r resulted in better outcomes than NVP-based ART in African children. Indeed, 39.6% of children in the NVP arm were in virological failure by study week 24, compared to 21.7% in the LPV/r arm (*p=*0.02). The cohort 2 of the P1060 trial showed that among children unexposed to prior nevirapine, the proportion of children who reached virological failure was significantly higher in the nevirapine group than in the LPV/r group (40.8% vs. 19.3%; *p*<0.001) [25]. However, children were highly different in terms of age at enrolment (younger in the P1060 cohort, 2 to 36 months versus 4 to186 months in our cohort), primary end-point (based on virological outcomes in the P1060 cohort versus clinical and immunological outcomes in our cohort), timing of ART outcome measures (24 weeks vs. 12 months on our cohort) and no viral load monitoring available in West Africa. Second, the P1060 study was conducted within a clinical trial with a random allocation of the LPV/r- or NVP-based first-line treatment whereas children on LPV/r first-line regimen in the West African cohort were more severely ill at baseline. Our study presents the ART response of HIV-infected children treated under the routine circumstances in West Africa which are not strictly comparable to the P1060 cohort.

Finally, in the Neverest randomized trial [16], children with prior exposure to sd-NVP, who initiated LPV/r-based treatment and achieved viral suppression (<400 copies/ml) for three or more months, where randomized to either remain on LPV/r or switch to NVP. The reuse of a NVP-based regimen after achieving viral suppression with a LPV/r-based regimen resulted in lower rates of viremia greater than 50 copies/ml (Kaplan-Meier probability, 0.4; 95% CI, 0.3–0.5) than maintaining a LPV/r regimen (0.6; 95% CI, 0.5–0.7), *p=*0.02.

Despite our adjusted analysis, the difference observed between the three groups in the occurrence of treatment failure could also be partially explained because PMTCT-exposed infants at ART initiation had earlier access to care while they had fewer clinical events and were more often treated by a PI-based regimen than the unexposed and unknown exposure children. Thus, they were supposed to have a better response to treatment than the unexposed children and the unknown ones who were more advanced in the disease with a worse prognosis at ART initiation.

This study highlights once again the importance of reducing the delay to ART access for HIV-infected children in resource limited countries. Indeed, these children often began treatment at more than five years of age, an advanced stage of disease, with severe malnutrition and immunosuppression [20,26–31].

The high level of treatment failure observed in this cohort raises many questions on the sensitivity and specificity of WHO 2010 criteria of paediatric ART treatment failure in resource limited countries, where viral load monitoring is rare. The kinetic of weight, CD4% recovery and of the decline of viral load could be more objective end-points even in the operational context [32]. This question was recently investigated in the ARROW trial showing that ART can be given across childhood using a CD4 monitoring that provided clinical benefit after the first year on ART compared to laboratory monitoring [33]. This needs to be further investigated.

Several methodological limitations were also observed in our study. Data used were collected under routine circumstances in Mali and Côte d’Ivoire, where viral load monitoring was not routinely assessed. The lack of virological testing limits the interpretation of our findings. It is well known that virological failure precedes clinical failure (sometimes by several months) and thus 12-month duration of follow-up without virological parameters can be misleading. Misclassification has also been well described with discordance between clinical or immunological and virological success following ART [34–38].

Our study also shows the difficulty of collecting retrospectively data on children’s PMTCT exposure, depending on the quality of medical records (existence, completeness, and accuracy). Indeed, although PMTCT interventions raise many questions on antiretroviral drugs resistance and clinical outcomes in ART-treated children, the PMTCT exposure is still infrequently documented in medical records in low- and middle-income countries. In numerous countries, the number of HIV-infected women and children who received antiretroviral for PMTCT is still unknown or grossly estimated [39–41]. Indeed, in our study, PMTCT exposure has not been correctly documented in medical records of more than half of children in care.

Children with unknown PMTCT exposure differed from others on baseline characteristics, but were more similar to the unexposed children than the exposed ones. They were included in our analysis of the treatment response in order to present a real-life experienced cohort and to reduce the selection biases. The lack of data on PMTCT exposure could be explained first by the long delay between birth of HIV-infected children and their first contact with a paediatrician. Second, PMTCT information was usually traced on hand-written maternal medical records and was not electronically recorded. Third, there was no systematic and organized linkage between children’s and maternal records.

This lack of data completeness has important implications for the use and interpretation of routine observational databases for research and audit, and highlights the need for regular data validation of these databases [42]. Different solutions to improve the quality of medical records and thus the health management system could be envisaged, such as implementing provider-based electronic medical records that improve the quality of data collected with a significant reduction in missing and incorrect information [43]. Improving data quality in the sub-Saharan HIV programmes and database that will inform important care providers’ decisions is crucial for the future.

## Conclusions

This study shows that in a context where only clinical and immunological monitoring were available, treatment failure in the first 12 months on ART often occurred in children younger than five years. Within six months, close to 50% of PMTCT-exposed children and two thirds of unexposed children were in treatment failure. Treatment failure after 12 months on ART was highly significantly associated with a severe immunodeficiency and AIDS clinical events at ART initiation. This highlights the need for an earlier initiation of ART in children, prior to the occurrence and detection of growth failure and severe immunosuppression [31,44,45]. Subsequent studies to identify predictors of growth and CD4 recovery and interventions to complement ART are needed to optimize health outcomes of HIV-infected children. Finally, the written documentation of the PMTCT exposure should be improved in order to better understand the impact of drug combinations on clinical and immunological responses, and survival in the operational context. This is of utmost importance as international guidelines move towards more complex drugs regimens for PMTCT [45].

Our study raises many questions on the success of paediatric ART in an operational context with a very long delay of ART initiation among children, the limited documentation of PMTCT exposure, the lack of biological monitoring, the insufficient linkage between maternal and child health services, and the insufficient human resources dedicated to paediatric HIV care. In parallel with increasing the access of children to ART, additional operational research is needed to optimize the quality of care of HIV-infected children in sub-Saharan Africa, and more especially in West Africa.
